# The Prolyl Hydroxylase Inhibitor Dimethyloxalylglycine Enhances Dentin Sialophoshoprotein Expression through VEGF-Induced Runx2 Stabilization

**DOI:** 10.1371/journal.pone.0112078

**Published:** 2014-11-04

**Authors:** Saeed Ur Rahman, Min-Sun Lee, Jeong-Hwa Baek, Hyun-Mo Ryoo, Kyung Mi Woo

**Affiliations:** 1 Department of Molecular Genetics, Dental Research Institute and BK21 Plus Program, School of Dentistry, Seoul National University, Seoul, Republic of Korea; 2 Department of Pharmacology & Dental Therapeutics, School of Dentistry, Seoul National University, Seoul, Republic of Korea; H. Lee Moffitt Cancer Center & Research Institute, United States of America

## Abstract

Prolyl hydroxylase (PHD) inhibitors are suggested as therapeutic agents for tissue regeneration based on their ability to induce pro-angiogenic responses. In this study, we examined the effect of the PHD inhibitor dimethyloxalylglycine (DMOG) on odontoblast maturation and sought to determine the underlying mechanism using MDPC-23 odontoblast-like cells. DMOG significantly enhanced matrix mineralization, confirmed by alizarin red staining and by measurement of the calcium content. DMOG dose-dependently increased alkaline phosphatase activity and the expressions of dentin sialophosphoprotein (Dspp) and osteocalcin. To determine the underlying events leading to DMOG-induced Dspp expression, we analyzed the effect of DMOG on Runx2. Knockdown of Runx2 using siRNAs decreased Dspp expression and prevented DMOG-induced Dspp expression. DMOG enhanced the transcriptional activity and level of Runx2 protein but not Runx2 transcript, and this enhancement was linked to the inhibitory effects of DMOG on the degradation of Runx2 protein. The vascular endothelial growth factor (VEGF) siRNAs profoundly decreased the Runx2 protein levels and inhibited the DMOG-increased Runx2 protein. Recombinant VEGF protein treatment significantly and dose-dependently increased the transcriptional activity and level of the Runx2 protein but not Runx2 transcript. Dspp expression was also enhanced by VEGF. Last, we examined the involvement of the Erk mitogen-activated protein kinase and Pin1 pathway in VEGF-enhanced Runx2 because this pathway can regulate the stability and activity of the Runx2 protein. VEGF stimulated Erk activation, and the inhibitors of Erk and Pin1 hampered VEGF-enhanced Runx2 protein. Taken together, the results of this study provide evidence that DMOG can enhance Dspp expression through VEGF-induced stabilization of Runx2 protein, and thus, suggest that DMOG can be used as a therapeutic tool for enhancing odontoblast maturation in dental procedures.

## Introduction

In treating the pathological conditions of the dentin-pulp complex, the regenerative dental procedures are a great challenge. Ideally, the therapeutic regimens must prevent the noxious stimuli of oral environment from entering the pulp, retain intact pulp tissue, and support the dentin formation [1]. Odontoblasts reside in dental pulp and responsible for dentin formation. Dentin is a calcified tissue that is similar to bone. The organic matrix of dentin consists of collagenous and non-collagenous proteins. Among the noncollagenous proteins, the cleavage products of dentin sialophosphoprotein (Dspp), dentin sialoprotein and dentin phosphoprotein, are contained at high levels in dentin [1]. Dspp is expressed predominantly in odontoblasts and at low levels in osteoblasts, and thus considered as a representative odontoblast marker gene [2]. Mutations of the human DSPP gene are associated with dentinogenesis imperfecta type II [3]. Dspp-deficient mice have teeth that display dentin mineralization defects, which are similar to those in human dentinogenesis imperfecta type III, indicating that Dspp plays a critical role in odontoblast differentiation and dentinogenesis [4].

Prolyl hydroxylases (PHDs) catalyze the prolyl hydroxylation of hypoxia-inducible factor (HIF)-1α in the presence of molecular oxygen, 2-oxoglutarate (2-OG), and Fe^2+^, which leads to the degradation of HIF-1α [5], [6]. HIF-1, which is a heterodimer composed of an oxygen-sensitive subunit (HIF-1α) and a constitutive subunit (HIF-1β), acts as the major transcriptional activator that regulates gene expression in cellular responses to low oxygen. The pharmacologic inhibition of PHDs mimics a hypoxic condition that stabilizes HIF-1. Accumulating evidence has indicated that triggering hypoxia-induced responses by PHD inhibitors improves tissue regeneration in animal models. PHD inhibitors markedly improved the defective wound healing process in diabetic and aged mice [7], [8]. Similar to PHD inhibitor effects on wound healing in soft tissues, PHD inhibitors increased bone fracture healing in mice [9]. Thus, the inhibition of PHDs has been suggested as a therapeutic strategy for the improvement of wound healing and tissue regeneration [10], [11].

Among hypoxia-induced responses, vascular endothelial growth factor (VEGF) and its effects on angiogenesis are well understood. Angiogenesis is considered essential for tissue regeneration because the vasculature provides a source of nutrients, oxygen, metabolic substrates, and access for circulating cells that help to support tissue regeneration [12]. VEGF promotes the proliferation of endothelial cells [13] and stimulates the elongation, network formation, and branching of nonproliferating endothelial cells in culture that are deprived of oxygen and nutrients [14]. In addition to the effects of VEGF on angiogenesis and the following supportive effects, VEGF can support neurogenesis and osteogenesis, at least in part, through its effects on neuroprogenitor cells and on bone cells [15], [16], while the mechanisms are not understood well.

It was reported that recombinant VEGF promoted the revascularization of severed human dental pulps [17]. The dental pulp cells exposed to hypoxic conditions or treated with PHD inhibitors increased VEGF production which led to pro-angiogenic responses in a cell-type specific manner [18], [19]. Thus, PHD inhibitor-stimulated VEGF may provide favorable environments for the regeneration of dentin-pulp complex by enhancing the angiogenic potential. Even though the possibility of applying PHD inhibitors to the dental procedures has been proposed, there is little evidence on that PHD inhibitors promote the regeneration of dentin-pulp complex, and the cellular and molecular events occurred by PHD inhibitors are largely unknown. Understanding the possible influences of PHD inhibitors on dentin-pulp complex is prerequisites for the improvement of the regenerative dental procedures using PHD inhibitors. In this study, we examined the effect of the PHD inhibitor dimethyloxalylglycine (DMOG) on odontoblast differentiation (maturation) in MDPC-23 odontoblast-like cells [20]. Furthermore, we sought to determine the mechanism by which DMOG enhances odontoblast maturation.

## Materials and Methods

### Cell culture

MDPC-23 cells [20] were maintained in Dulbecco's modified Eagle's medium (DMEM) (Hyclone, Logan, UT, USA) supplemented with 10% heat-inactivated fetal bovine serum (FBS) (Gibco BRL, Grand Island, NY, USA). To induce odontoblast differentiation, we cultured the cells in a differentiation medium (growth medium supplemented with 10 mM β-glycerophosphate and 50 µg/ml ascorbic acid) in the presence or absence of DMOG (Cayman Chemical, Ann Arbor, MI, USA) or of recombinant murine VEGF (PeproTech, Rocky Hill, NJ, USA) for the indicated periods. Cell viability was determined using a Cell Counting Kit-8 (CCK-8) (Dojindo Laboratory, Kumamoto, Japan).

### Calcium deposition assay

Cells were seeded at a density of 5×10^4^ cells per well in 24-well plates and cultured in the differentiation medium for 7 days. The calcium content was measured using a calcium assay kit (QuantiChrom™ Bioassay Systems, Hayward, CA, USA) according to the manufacturer's instructions.

### Alkaline phosphatase activity

Cells were seeded at a density of 5×10^4^ cells per well in 24-well plates and cultured in the differentiation medium for 4 days. The alkaline phosphatase activity was measured using *p*-nitrophenyl phosphate (*p*-NPP) as a substrate, as described previously [21].

### Extraction of total RNAs and RT-qPCR

The cells were cultured in the differentiation medium and harvested. Total RNAs were extracted using RNA iso Plus (Takara, Otsu, Japan) reagents, and cDNA was synthesized using a PrimeScript RT reagent kit (Takara). Quantitative real-time PCR was performed on a Real-time PCR system (Applied Biosystems, Foster City, CA, USA). Glyceraldehyde-3-phosphate dehydrogenase (GAPDH) expression was used as a control for normalizing the expression data. The primer sequences used in this study are listed in Table 1.

**Table 1:** Sequences of primers used in RT-PCR.

Molecules	Primer sequences	Accession No. (GenBank)
Runx2	5′- TTCTCCAACCCACGAATGCAC-3′	NM_001146038.
	5′-CAGGTACGTGTGGTAGTGAGT-3′′	
Vegf	5′- CCACGTCAGAGAGCAACATCA-3	NM_009505.1
	5′- TCATTCTCTCTATGTGGCTTTT-3′	
Dspp	5′- AACACATCCAGGAACTGCAGCACA-3′	NM_010080.2
	5′- TGACTCGGAGCCATTCCCATCTCT-3′	
Osteocalcin	5′- CTGAGTCTGACAAAGCCTTC-3′	NM_007541.3
	5′- GCTGTGACATCCATACTTGC-3′	
H3f3	5′- CTGCGCTTCCAGAGTGCAGCT-3′	NM_008210.4
	5′- AGCACGTTCTCCGCGTATGCG-3′	
Gapdh	5′- AGGTCGGTGTGAACGGATTTG-3′	NM_001289726.1
	5′- TGTAGACCATGTAGTTGAGGTCA-3′	

### Western blot analysis

MDPC-23 cells were seeded at a density of 5×10^5^ cells per 60-mm culture dish and cultured in the differentiation medium for 2 days. Whole cell lysates were prepared, and Western blot analyses were performed to detect HIF-1α, VEGF, Runx2, pERK, and ERK, as described previously [21]–[23]. The following primary antibodies were used: monoclonal anti-Runx2 protein (MBL, Woburn, MA, USA), monoclonal anti-VEGF (Abcam, Cambridge, MA, USA), monoclonal anti-HIF-1α (Novus Biologicals, Littleton, CO, USA), polyclonal anti-pERK (Cell Signaling, Danvers, MA, USA), and polyclonal anti-ERK (Cell Signaling). Anti-β-actin HRP-conjugated mouse monoclonal IgG_1_ antibody (Santa Cruz Biotechnology, Santa Cruz, CA, USA) was used for the loading control. The band intensities were measured by the softeware ImageJ (NIH, USA), and relative protein levels were normalized by β-actin.

### Knockdown with siRNAs

siRNAs were used to knockdown the expression of Vegf or of Runx2. The siRNA oligonucleotides against Vegf and Runx2 (siGENOME SMARTpool) were purchased from Dharmacon (Lafayette, CO, USA). Scrambled siRNA (siGENOME Non-Targeting siRNA) was used as a control. MDPC-23 cells were seeded at a density of 5×10^5^ cells per 60-mm culture dish and transfected with each specific siRNA in accordance with the manufacturer's instructions.

### Reporter assay

MDPC-23 cells were seeded on 96 wells tissue culture plates at a density of 5×10^3^ cells per well. The Runx2 activity reporter vector (6XOSE2-Luc) or the empty PGL3 basic vector was transfected into MDPC-23 cells using Genefectine (Genetrone, Gwangmyung, Korea). After 36 h of transfection, the cells were treated with different concentrations of VEGF or DMOG for an additional 3–24 h. The luciferase activity was measured using a Dual-Glo Luciferase Assay System (Promega, Madison, WI, USA). Renilla luciferase activity was used for normalizing the data.

### Statistical analysis

All data are presented as the mean ± standard deviation (SD). A statistical analysis was performed using Student's t-test. The difference between groups was considered significant when the *p*-value was less than 0.05.

## Results

**Figure 1:**
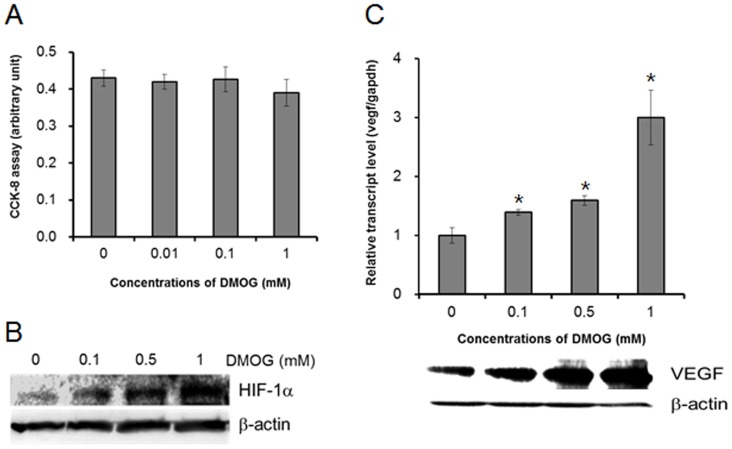
DMOG induces pro-angiogenic responses without cell toxicity in MDPC-23 cells. (A) MDPC-23 cells were incubated for 24 h in the presence or absence of DMOG, and cytotoxicity was evaluated. DMOG does not induce cytotoxic effects at concentrations lower than 1 mM in MDPC-23 cells. The data represent the mean ± SD of quadruplicates. (B, C) MDPC-23 cells were incubated for 24 h in the presence or absence of DMOG, and western blot analyses for HIF-1α (B) and VEGF (C, lower) and RT-qPCR for VEGF (C, upper) were performed. The data are presented as the mean ± SD of triplicates. *Significantly different from control (Student's t-test, *p*<0.05).

**Figure 2:**
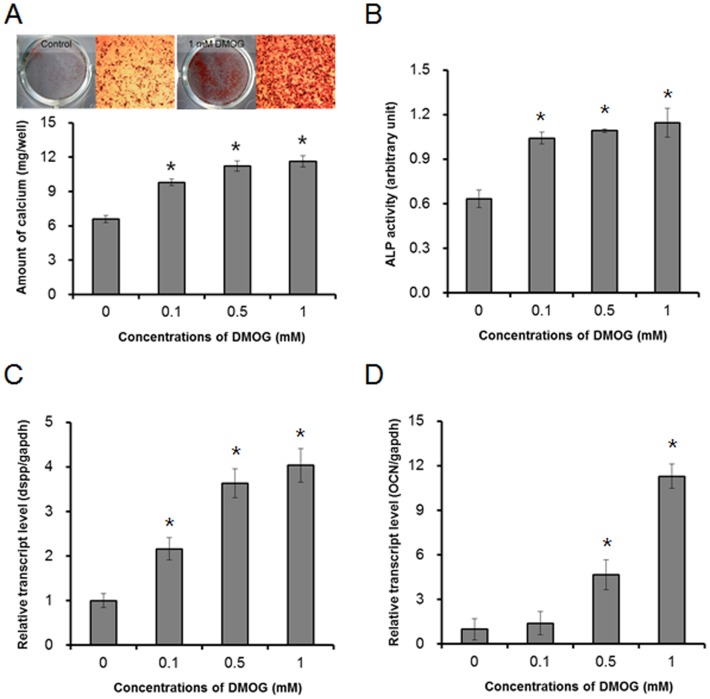
DMOG enhances the odontoblast maturation of MDPC-23 cells. The cells were cultured in the differentiation medium for 7 days (A) or for 4 days (B–D) in the presence or absence of DMOG. (A) Matrix mineralization was examined by alizarin red S staining (upper) and by calcium deposition (lower). (B) ALP activity was determined from total cell lysates, and (C, D) the expression of Dspp and osteocalcin, which are odontoblast differentiation markers, was measured by quantitative RT-PCR. The data are presented as the mean ± SD of triplicates. *Significantly different from control (Student's t-test, *p*<0.05).

Before evaluating the effect of DMOG on odontoblast maturation, we examined whether DMOG shows cytotoxicity in MDPC-23 cells. After treatment for 24 h, DMOG did not exhibit cytotoxic effects at the concentrations used in this study (Fig. 1A; ). Even when MDPC-23 cells were treated with 1 mM DMOG for 7 days under a differentiation condition with β-glycerophosphate and ascorbic acid, obvious cell death was not observed. Consistent with previous reports [19], DMOG increased the level of the HIF-1α and stimulated the expression of VEGF, which is a pro-angiogenic molecule under the control of HIF-1 (Figs. 1B, 1C). Then, we examined the effect of DMOG on the differentiation of MDPC-23 cells. MDPC-23 cells were cultured in a differentiation medium, and matrix mineralization was determined by alizarin red staining and by the extent of calcium deposition. DMOG significantly enhanced matrix mineralization in a dose-dependent manner (Fig. 2A). To verify that the observed DMOG-enhanced matrix mineralization is associated with odontoblast differentiation, we examined alkaline phosphatase activity and the mRNA expression levels of odontoblast differentiation marker genes. Consistent with the alizarin red staining and calcium deposition results, DMOG dose-dependently increased alkaline phosphatase activity and the mRNA levels of Dspp and of osteocalcin (Figs. 2B, 2C, and 2D; Table S3). These results indicate that DMOG stimulates matrix mineralization through enhancing the differentiation of MDPC-23 cells.

**Figure 3:**
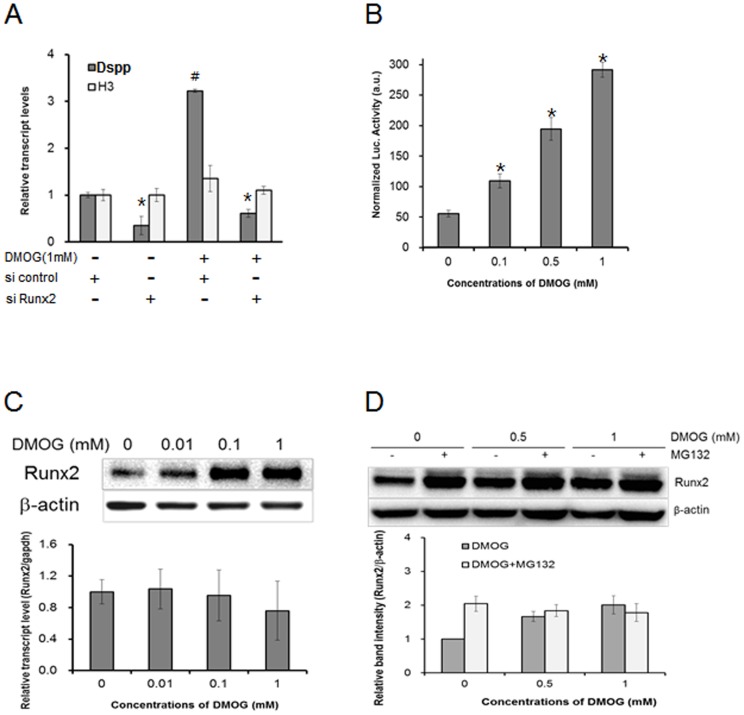
DMOG increases the level and transcriptional activity of the Runx2 protein, leading to Dspp expression in MDPC-23 cells. (A) Runx2 knockdown suppresses DMOG stimulation of Dspp expression. The cells were transiently transfected with Runx2 siRNA or with control siRNA and incubated in the presence or absence of 1 mM DMOG for 48 h. The data are presented as the mean ± SD of triplicates. *Significantly different from control siRNA. ^#^Significantly different from none-DMOG treated control (Student's t-test, *p*<0.05). (B) Transcriptional activity of Runx2. The Runx2 reporter 6XOSE-Luc was transfected into MDPC-23 cells, and the cells were cultured for 48 h. Runx2 reporter luciferase activity was measured and normalized by Renilla luciferase activity. The data are presented as the mean ± SD (n = 6). *Significantly different from control (Student's t-test, *p*<0.05). (C) The levels of Runx2 protein (upper) and transcripts (lower). (D) The cells were treated with 2 µM of MG132 for 24 h in the presence or absence of DMOG, and a western blot analysis was performed for Runx2. Western blot quantification was performed by measuring bands intensities, and the relative levels were normalized by β-actin.

To determine the molecular mechanism by which DMOG enhances odontoblast maturation, we focused on the regulation of Dspp expression because Dspp is a representative odontoblast marker gene [2]–[3]. Runx2, which is an essential transcription factor for bone and tooth development, can control the expression of mineralization-associated genes, including Dspp [24]. Thus, we first examined the effect of Runx2 on Dspp expression in MDPC-23 cells through knockdown of Runx2 expression. Runx2 transcript and protein levels were efficiently reduced by Runx2 siRNA, whereas Runx2 siRNA did not alter the expression of histone H3 as an internal standard, confirming the specificity of the Runx2 siRNA we used. Runx2 siRNA significantly decreased Dspp expression in MDPC-23 cells. Furthermore, knockdown of Runx2 prevented DMOG stimulation of Dspp expression (Fig. 3A). These results demonstrate that Runx2 plays a role as a mediator for DMOG stimulation of Dspp expression. DMOG treatment significantly increased the Runx2 protein level in a dose-dependent manner (Fig. 3C), whereas the Runx2 transcript level was not significantly altered by DMOG. Runx2 transcriptional activity was also analyzed using a Runx2 reporter assay with 6XOSE2, which has six tandem repeats of the osteoblast-specific *cis*-element. The cells treated with DMOG exhibited up to approximately five-fold higher levels of Runx2 activity after the activity was normalized by Renilla luciferase activity (Fig. 3B; Table S2). The Runx2 activity corresponded to the level of the Runx2 protein. However, because a discrepancy between the levels of the Runx2 transcript and protein was observed, the degradation of Runx2 protein was examined. The proteasome inhibitor MG132 increased Runx2 protein levels in MDPC-23 cells, regardless of DMOG treatment (Fig. 3D). The Runx2 protein levels were not significantly altered by DMOG in the presence of MG132. The results indicate that Runx2 protein was substantially degraded in a proteasome-dependent manner and that DMOG prevented Runx2 protein from degradation in MDPC-23 cells. Taken together, these findings suggest that DMOG may up-regulate Dspp expression by enhancing Runx2 protein stabilization.

**Figure 4:**
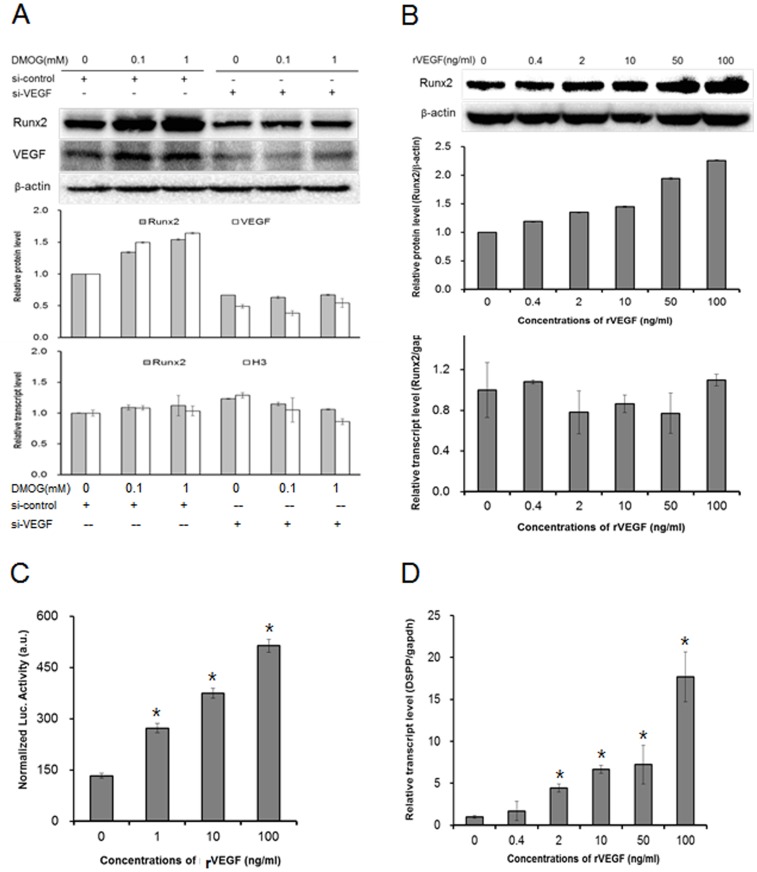
DMOG-induced VEGF stabilizes the Runx2 protein in MDPC-23 cells. (A) DMOG-enhanced Runx2 protein is VEGF-dependent. MDPC-23 cells were transiently transfected with Vegf siRNA or with control siRNA and incubated in the presence or absence of DMOG for 48 h. Western blots for Runx2 protein and for VEGF and RT-qPCRs for Runx2 and for Histone H3 were performed. Western blot quantification was performed by measuring bands intensities, and the relative levels were normalized by β-actin. (B) Recombinant VEGF increases the Runx2 protein levels (upper) but not Runx2 transcripts (lower). MDPC-23 cells were treated with VEGF (50 ng/ml), and a western blot and RT-qPCR were performed. Western blot quantification was performed by measuring bands intensities, and the relative levels were normalized by β-actin. (C) Transcriptional activity of Runx2 upon VEGF treatment. The Runx2 reporter 6XOSE-Luc was transfected into MDPC-23 cells, and the cells were cultured for 48 h in the presence or absence of VEGF. Runx2 reporter luciferase activity was measured and normalized by Renilla luciferase activity. The data are presented as the mean ± SD (n = 6). *Significantly different from control (Student's t-test, *p*<0.05). (D) MDPC-23 cells were treated with VEGF for 24 h, and RT-qPCR was performed for Dspp expression. The data are presented as the mean ± SD (n = 3). *Significantly different from control (Student's t-test, *p*<0.05).

A previous report demonstrated that mice with conditional Vegf deficiency in osteoblastic precursor cells exhibited an osteoporosis-like phenotype and that reduced Runx2 transcriptional activity was observed in mesenchymal stem cells with reduced Vegf expression [16]. Based on this report and our data that DMOG increased VEGF expression in MDPC-23 cells (Fig. 1C), we hypothesized that DMOG-induced VEGF causes an increase in the Runx2 protein in MDPC-23 cells. To examine the role of Vegf in DMOG stimulation of Runx2 protein stabilization and Dspp expression, we knocked down the expression of Vegf using siRNA. The Vegf transcript and protein levels were efficiently reduced by Vegf siRNA, whereas the expression of histone H3 was not significantly altered. Vegf siRNA profoundly decreased the Runx2 protein levels, whereas the Runx2 transcript levels were not significantly altered by Vegf siRNA, suggesting that VEGF induces the inhibition of Runx2 protein degradation. Furthermore, Vegf knockdown prevented DMOG-enhanced Runx2 protein expression (Fig. 4A). These results indicate that DMOG-induced VEGF led to Runx2 protein stabilization. Then, we treated the culture with recombinant VEGF protein and confirmed the effect of VEGF on Runx2 levels and on its transcriptional activity. VEGF treatment significantly increased the Runx2 protein level in a dose-dependent manner (Fig. 4B), whereas the Runx2 transcript level was not significantly altered by VEGF. Runx2 transcriptional activity was also increased dose-dependently by VEGF and corresponded to the Runx2 protein level (Fig. 4C). Furthermore, VEGF increased Dspp expression in MDPC-23 cells (Fig. 4D). These results demonstrate that VEGF stimulation increases the Runx2 protein level, contributing to Dspp expression. To our best knowledge, this report is the first demonstrating that VEGF can stabilize Runx2 protein and enhance Dspp expression in the cells of odontogenic lineage.

**Figure 5:**
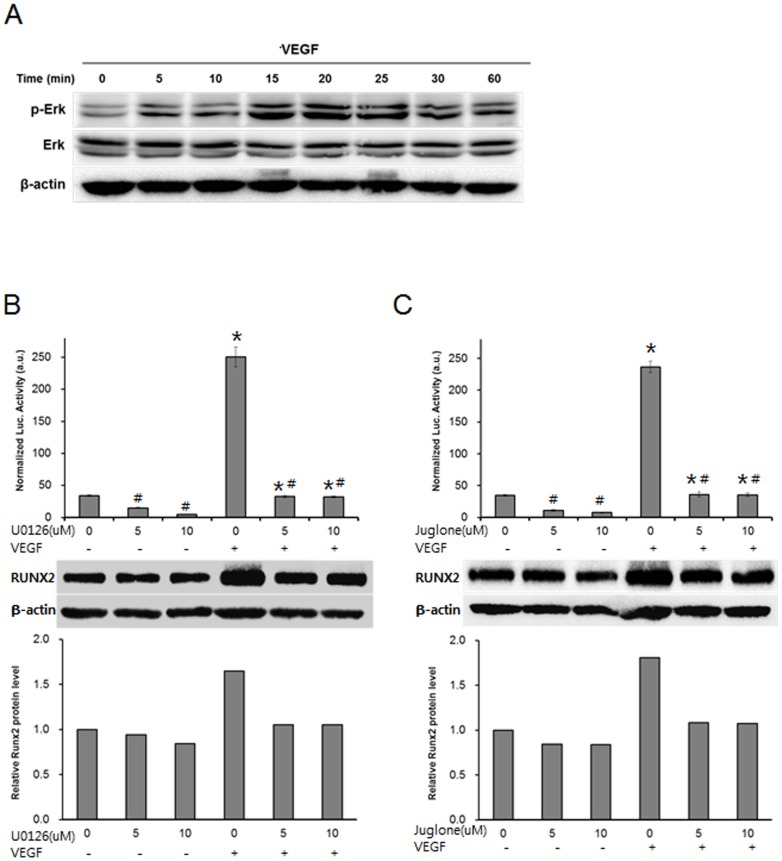
The ERK/PIN1 pathway is involved in VEGF-enhanced Runx2 protein levels in MDPC-23 cells. (A) The cells were cultured in the presence or absence of recombinant VEGF (50 ng/ml) for the indicated times and subjected to western blot analyses for pErk and for Erk. (B, C) The Runx2 reporter 6XOSE-Luc was transfected into MDPC-23 cells, and the cells were incubated for 36 h. The cells were further treated with U0126 (B) or with juglone (C) in serum-free media for 1 h before recombinant VEGF treatment, and then the cells were cultured in the presence or absence of recombinant VEGF (50 ng/ml) for an additional 3 h. Then, the cultures were subjected to luciferase activity tests (upper) and western blot analyses (lower). Western blot quantification was performed by measuring bands intensities, and the relative levels were normalized by β-actin. The luciferase data are presented as the mean ± SD (n = 3). *Significantly different from TCD (Student's t-test, *p*<0.05). ^#^Significantly different from control (Student's t-test, *p*<0.05).

Last, we tested the involvement of Erk and Pin1 in the VEGF-enhancement of the Runx2 protein in MDPC-23 cells because previous reports showed that Erk activation and consequent Pin1 modified the Runx2 protein in FGF-stimulated osteoblasts, led to Runx2 protein stabilization, and enhanced its transcriptional activity [22], [23]. We analyzed Erk phosphorylation upon VEGF stimulation, and the effects of the inhibitors on the Runx2 protein level and transcriptional activity were evaluated. MDPC-23 cells were cultured in serum-free media for 3 h before VEGF stimulation. VEGF clearly began to increase the level of the phosphorylated form of the Erk as early as 5 min after stimulation (Fig. 5A). Erk phosphorylation peaked at 15∼20 min, reduced afterward, and maintained at a slightly higher level than at the starting point. The inhibitor U0126, which restrains Erk phosphorylation, reduced the Runx2 protein level and its transcriptional activity and abrogated VEGF-stimulated Runx2 increase (Fig. 5B). Similar to U0126, juglone, which inhibits Pin1 isomerase activity, decreased the Runx2 protein level and transcriptional activity and prevented VEGF-induced Runx2 protein increase (Fig. 5C). These results indicate that VEGF regulates the Runx2 protein level and transcriptional activity via Erk and Pin1 activation.

## Discussion

In this study, we demonstrated that the PHD inhibitor DMOG enhances the differentiation of murine dental papilla-derived, odontoblast-like MDPC-23 cells, as confirmed by the extent of calcium deposition, alizarin red staining, ALP activity, and dentin-matrix protein expression. DMOG also enhances the level and transcriptional activity of Runx2 protein, leading to Dspp expression by DMOG-induced VEGF regulation of Runx2 protein degradation. Additionally, VEGF treatment increases the level of Runx2 protein and transcriptional activity, which led to Dspp expression. Furthermore, VEGF stimulates Erk activation, and inhibitors of Erk and Pin1 hamper VEGF-induced Runx2 protein stabilization. These results provide evidence that DMOG can directly enhance odontoblast maturation, in addition to its well-known effect on angiogenesis.

Previous studies suggest that VEGF can stimulate osteoblast differentiation [25]–[27]. In addition, it was proposed that intracellular VEGF regulates osteoblast differentiation through Runx2 [16]; however, the mechanism by which VEGF increases Runx2 was unknown. In this study, we provide evidence that VEGF increases the Runx2 protein by preventing Runx2 protein degradation. Additionally, VEGF-induced Erk activation may be involved in Runx2 protein stabilization as FGF2-stimulated Erk activation led to Runx2 protein stabilization [22], [23]. Combined with the reports that Runx2 can serve as a transcription factor to induce Vegf transcription [28], [29], our results support the existence of a positive feedback loop between VEGF and Runx2 protein, which can synergistically control dentinogenesis (or osteogenesis) and angiogenesis.

Runx2, which is required for osteoblast differentiation and for bone development [30], [33], is also essential for tooth development. Dental abnormalities can be found in the human syndrome cleidocranial dysplasia, which occurs due to haploinsufficiency caused by Runx2 mutations [31]–[34]. Runx2 knockout mice exhibit an arrest of molar tooth development at the early cap stage, suggesting a requirement for Runx2 in the progression of tooth development from the cap stage to the bell stage [35]. Tooth development was examined in Runx2 transgenic mice under the control of Col1α1, and the transgenic mice showed the altered morphology of odontoblasts and down-regulation of dentin matrix protein genes [36] Currently, Runx2 is thought to play a stage-specific role in the lineage determination and differentiation of odontoblasts from dental papilla mesenchyme during tooth development, based on studies of the expression pattern, the knockout and targeted mice, and forced over-expression of Runx2 into odontoblast cell lines [24], [36], [37]. Runx2 protein was expressed in odontoblast-like cells during reparative dentin formation [38] and in dentin-like tissues after tooth replantation [39]. Expression of Runx2 was up-regulated during odontoblastic differentiation of human pulp-derived cells [38], [40], [41]. These reports suggest that Runx2 may play a role in the processes of dentin repair or regeneration. In this study, we first examined the effect of Runx2 on Dspp expression in MDPC-23 cells by silencing the expression of Runx2. The Runx2 siRNA significantly decreased Dspp expression in MDPC-23 cells. Furthermore, knockdown of Runx2 prevented DMOG stimulation of Dspp expression. These results demonstrate that a pharmacological agent, DMOG, can modulate Runx2 protein to enhance Dspp expression and suggest that DMOG can be used in the procedures for enhancing the odontoblast differentiation during reparative dentin formation.

Small molecules are appealing for regenerative medicine applications because many exhibit extended in vivo stability, limited periods of action, low cost, and scalable production [12]. In these applications, biomaterials can provide platforms to deliver therapeutic small molecules to injury sites. PHD inhibitors are suggested as alternatives to angiogenic growth factors for enhancing the healing of soft and hard tissues [11], [12]. The PHD inhibitor DMOG is a 2-OG analog that may work by competitively inhibiting the 2-OG binding site in PHDs and factors-inhibiting HIF enzymes that regulate HIF-1α. The pan-hydroxylase inhibitors, such as DMOG, have been shown to reduce inflammation in animal models of colitis, infection, and sepsis [42]–[44]. Considering the inflammatory features of pulp pathologies, DMOG was chosen in this study. We showed that DMOG enhances matrix mineralization and Dspp expression in MDPC-23 cells in this study and also observed the similar effects of DMOG in human dental pulp stem cells (Figure S1). Given that MDPC-23 cells are considered as odontoblast-like cells [20], DMOG can promote the differentiation of dental pulp stem cells to odontoblasts and further enhance the differentiation. It implicates that DMOG can be used to promote reparative and reactionary tertiary dentin formation as well as to regenerate the dentin-pulp complex. Tertiary dentin is formed in response to external stimuli via exposed dentinal tubules. As suggested previously [45], topical application of the biologic factor DMOG to permeate across dentin to modify the formation of tertiary dentin may lower dentin sensitivity and protect the pulp. Although further *in vivo* studies with the proper biomaterials as a vehicle are required, our results together with previous reports in the literature suggest that DMOG can be efficiently used as a therapeutic tool for enhancing odontoblast maturation in dental procedures.

## Supporting Information

Figure S1 **Effect of DMOG on human dental pulp cells.** DMOG promotes odontoblastic differentiation of human dental pulp cells. (A) Alkalinephosphatase staining. (B) Expression of Dspp. Human dental pulp cells were cultured for 7 days in the absence (control) or presence (DMOG) of 100 mM DMOG in a differentiation medium * Significantly different from control (p<0.01).
(PDF)

Table S1 **Dataset for qPCR.** Dataset of quantitative real time PCR analysis to determine the expressions of VEGF, DSPP, OCN, and Runx2 genes.
(XLSX)

Table S2 **Dataset for luciferase assay.** Dataset of Runx2 transcriptional activity was analyzed using Runx2 reporter assay with 6XOSE2 construct plasmid.
(XLSX)

Table S3 **Dataset for etc.** The dataset of cytotoxicity, calcium, and ALP activity assay analysis.
(XLSX)
