# Supplementary material for: The Prolyl Hydroxylase Inhibitor Dimethyloxalylglycine Enhances Dentin Sialophoshoprotein Expression through VEGF-Induced Runx2 Stabilization
Source: PLoS One. 2014 Nov 4;9(11):e112078. doi: 10.1371/journal.pone.0112078 (PMC4219688; doi:10.1371/journal.pone.0112078)
Supplement: Figure S1 — Effect of DMOG on human dental pulp cells. DMOG promotes odontoblastic differentiation of human dental pulp cells. (A) Alkalinephosphatase staining. (B) Expression of Dspp. Human dental pulp cells were cultured for 7 days in the absence (control) or presence (DMOG) of 100 mM DMOG in a differentiation medium * Significantly different from control (p<0.01). (PDF) [file pone.0112078.s001.pdf]

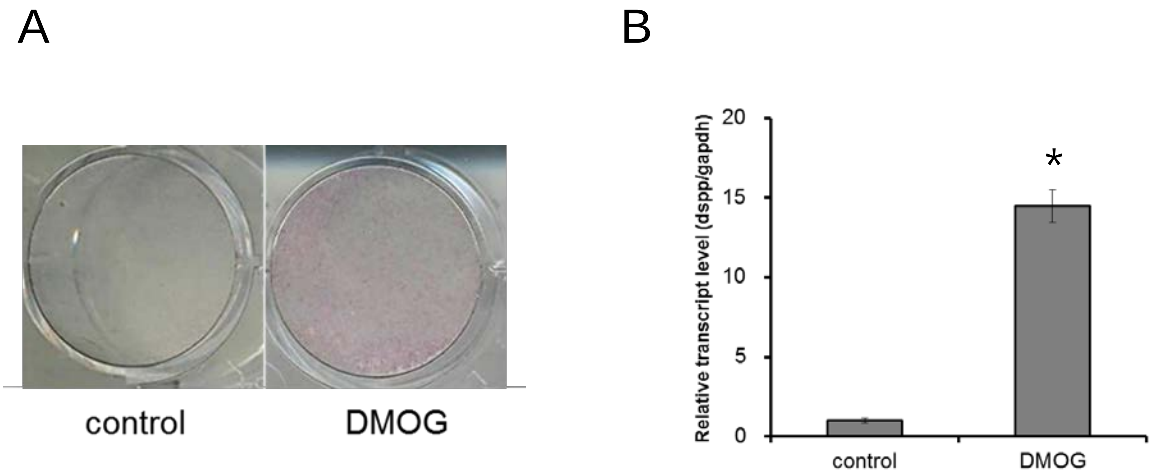

Figure S1. DMOG promotes odontoblastic differentiation of human dental pulp cells. (A) Alkaline phosphatase staining. (B) Expression of Dspp. Human dental pulp cells were cultured for 7 days in the absence (control) or presence (DMOG) of 100 μM DMOG. in a differentiation medium * Significantly different from control ($p<0.01$).
